# Impact of preoperative anemia on postoperative rate of severe complications and short-term clinical outcomes after retroperitoneal tumor resection

**DOI:** 10.1186/s12871-025-03573-2

**Published:** 2025-12-27

**Authors:** Subing Guo, Hua Zhang, Dongyang Ma, Haoyao Dong, Yongjie Chen, Bo Wang, Lan Yao, Kunpeng Liu, Yi Liu

**Affiliations:** 1 Department of Anesthesiology, Shanxi Medical University, No.56 Xinjian South Road ,Yingze District,Taiyuan City, ShanXi, 030000 China; 2 Department of Anesthesiology, Peking University International Hospital, No.1 Shengmingyuan Road,Life Science Park, Changping District, Beijing, 102206 China; 3 Department of Anesthesiology, PKUCare Rehabilitation Hospital, No. 8 Shengmingyuan Road, Life Science Park, Changping District, Beijing, 102206 China; 4 The Clinical Epidemiology Research Center, Peking University Third Hospital, No. 49 Huayuan North Road, Haidian District, Beijing, 100096 Beijing China; 5 Department of Anesthesiology, Cancer Hospital Affiliated to Shanxi Medical University, Taiyuan City, China

**Keywords:** Preoperative anemia, Retroperitoneal tumors, Complications, Prognosis, Clavien-Dindo classification

## Abstract

**Objective:**

To investigate the impact of preoperative anemia incidence of severe complications and short-term clinical outcomes in patients undergoing retroperitoneal tumor resection.

**Methods:**

We performed a retrospective cohort study, enrolling patients with retroperitoneal tumors who received resection at Peking University International Hospital from January 2015 to December 2023. Patient baseline data were collected, and the following outcome measures were recorded:​

• The primary outcome measure was the grade of postoperative complications;

•The secondary outcome measures included duration of mechanical ventilation, length of ICU stay, postoperative hospital stay, total hospital stay, postoperative 24-hour mortality, and postoperative 30-day mortality.​

Patients were divided into an anemia group and a non-anemia group according to the World Health Organization (WHO) criteria for anemia to analyze the effect of preoperative anemia on the aforementioned indicators.

**Results:**

A total of 1968 patients were included in this study, among whom 1100 (465 males and 635 females) had preoperative anemia, with an incidence rate of 55.9%. Statistically significant differences were noted between the two groups in terms of gender, age, body mass index (BMI), American Society of Anesthesiologists (ASA) physical status classification, and number of previous surgeries (all *P* < 0.05). Specifically, the anemia group exhibited a higher proportion of female patients, older age, lower BMI, a higher percentage of patients with ASA class III or IV, more patients with two or more previous surgeries, longer operation duration, and longer anesthesia duration compared with the non-anemia group.

Compared to the non-anemia group, the anemia group had a higher incidence of severe postoperative complications, along with significantly prolonged duration of mechanical ventilation, length of ICU stay, postoperative hospital stay, and total hospital stay. In the entire cohort, 4 patients (0.2%) died within 24 hours postoperatively, and 20 patients (1.0%) died within 30 days postoperatively.

Multivariate analysis, after adjusting for confounding factors including gender, age, and BMI, revealed that patients in the preoperative anemia group had a significantly increased risk of severe postoperative complications, as well as significantly prolonged duration of mechanical ventilation, ICU stay, postoperative hospital stay, and total hospital stay. Preoperative anemia was not correlated with mortality at 24 hours or 30 days after surgery.

**Conclusion:**

Preoperative anemia is an independent risk factor for increased incidence of severe postoperative complications, as well as prolonged duration of mechanical ventilation, length of ICU stay, total hospital stay, and postoperative hospital stay in patients undergoing retroperitoneal tumor resection. Proactive management of preoperative anemia is critical for reducing postoperative complications and enhancing clinical outcomes in patients undergoing retroperitoneal tumor resection, thereby offering valuable insights for optimizing perioperative care in this population.

**Supplementary Information:**

The online version contains supplementary material available at 10.1186/s12871-025-03573-2.

Retroperitoneal tumors (RPTs) are relatively rare neoplasms that originate primarily in the retroperitoneal space, including the presacral and pelvic floor spaces [1]. Approximately 80% of RPTs are malignant; however, these malignant tumors typically exhibit relatively low malignancy, slow growth rates [2], and a low incidence of distant metastasis. Surgical resection remains the current mainstay of treatment [3, 4] .Owing to their unique anatomical location, achieving adequate surgical margins during resection is often challenging. Furthermore, RPTs are characterized by occult growth patterns and insensitivity to radiotherapy and chemotherapy, which contribute to a high tendency for recurrence—often necessitating multiple surgical procedures. Notably, the severity of anemia has been shown to be positively correlated with the number of tumor resections [5]. Given these factors, preoperative anemia in patients with RPTs is a clinical issue that warrants significant attention.

Cancer patients are at increased risk of anemia due to factors such as hypermetabolic consumption, tumor invasion of blood vessels or the gastrointestinal tract. This reduction in hemoglobin impairs oxygen-carrying capacity, leading to tissue hypoxia that can compromise immune function, delay tissue repair, and impair organ recovery. These effects adversely affect postoperative recovery and long-term outcomes, including shortened overall survival (OS) and disease-free survival (DFS) [6, 7].

Patients with preoperative anemia are more susceptible to intraoperative complications such as hypotension and arrhythmias. Additionally, anemia is associated with increased transfusion requirements, higher rates of postoperative complications [8], surgical site infections, prolonged hospital stays [9], and elevated mortality. It also impedes early rehabilitation and diminishes postoperative quality of life [7]. Therefore, preoperative anemia warrants heightened attention in clinical practice to improve perioperative outcomes.

Recent studies have established a association between preoperative anemia and poor outcomes in various surgical procedures, including lumbar spine [10], cardiac [11], and neurosurgical operations [12]. However, the impact of preoperative anemia on the prognosis of patients with retroperitoneal tumors (RPTs) remains unexplored. This study aimed to evaluate the effect of preoperative anemia on postoperative outcomes in RPT patients, including complication severity, length of hospital stay, and short-term survival rates. By examining the relationship between preoperative anemia and postoperative recovery, our findings seek to inform clinical strategies and ultimately improve treatment efficacy and prognosis in this patient population.

## Materials and methods

### Study design

This retrospective cohort study aimed to assess the influence of preoperative anemia on the severity of postoperative complications and short-term morbidity and survival outcomes in patients undergoing resection for retroperitoneal tumors.

### Study population

Consecutive patients who undergoing surgical resection for retroperitoneal tumors (RPTs) at Peking University International Hospital between January 2015 and December 2023 were initially considered for inclusion. Eligible participants were those aged 18 years or older who received elective surgical treatment for RPTs at our institution. Exclusion criteria included: postoperatively pathologically confirmed non-RPTs diagnoses, patients who undergoing emergency or unplanned surgery, and those with missing or incomplete medical records. The study protocol was reviewed and approved by the Ethics Committee of Peking University International Hospital (Approval No. 2022-KY-0032-01). Due to the retrospective nature of the study and the use of anonymized data, the requirement for informed consent was waived.

### Observation indicators

Based on the WHO criteria for anemia, patients were categorized into two groups: non-anemic (hemoglobin ≥ 130 g/L in men and ≥ 120 g/L in non-pregnant women) and anemic (hemoglobin < 130 g/L in men and < 120 g/L in non-pregnant women).(The preoperative hemoglobin of the patients was based on the data from the last preoperative blood routine. As this was a retrospective study, the types of anemia could not be distinguished in detail.)The following baseline and perioperative variables were collected: age, gender, height, weight, body mass index (BMI), number of previous surgeries, and preoperative hemoglobin level. Intraoperative parameters included estimated blood loss, transfusion volume, fluid balance, duration of surgery, and anesthesia time. The primary outcome was the, incidence of severe complications (number of people with severe complications after surgery/total number of data) graded according to the Clavien–Dindo (CD) classification system [13]. Complications were defined as mild (CD grade I–II) or severe (CD grade ≥ III). Secondary outcomes comprised duration of mechanical ventilation, length of ICU stay, postoperative hospital stay, total hospitalization time, as well as 24-hour and 30-day mortality after surgery. Common complications following radical resection of retroperitoneal tumors—such as abdominal infection, hemorrhage, enteric fistula, pancreatic leak, gastroparesis, intestinal obstruction, pleural effusion, hypoproteinemia, and death—were documented and systematically evaluated (see Appendix Table 1, and 2 for details).

### Statistical analysis

Statistical analysis was performed using R software version 4.0.3. Continuous variables were tested for normality using the Shapiro test; those conforming to a normal distribution were expressed as mean ± standard deviation (x̄±s), and those not conforming were expressed as median (min, max). Categorical variables were expressed as frequency (%). The Mann-Whitney U test was used for continuous variables with non-normal distributions; the chi-square test or Fisher’s exact test was used for categorical data such as complication severity. Multivariate analysis of postoperative complications was performed using logistic regression analysis. Multivariate analysis of total hospital stay was performed using multiple linear regression. Factors with *P* < 0.05 in univariate analysis were included in the regression analysis. Variables were selected for the multivariate model based on clinical significance and univariate analysis results (*P* < 0.05), and independent influencing factors were screened using the backward method. A two-sided *P* < 0.05 was considered statistically significant.

## Results

### Basic patient characteristics

A total of 2035 patients who undergoing surgical resection for retroperitoneal tumors were initially screened. After excluding 20 patients with non-retroperitoneal tumors, 3 emergency cases, 39 patients under 18 years of age, and 5 patients with incomplete data, 1968 patients were included in the final analysis. The cohort consisted of 913 males (46.4%) and 1055 females (53.6%), with a median age of 51 years old (range from 18 to 94). Based on WHO criteria for anemia, preoperative anemia was identified in 1100 patients (465 males and 635 females), corresponding to an overall anemia rate of 55.9% Table 1.

**Table 1:** Baseline characteristics of patients with and without preoperative anemia data are presented as median (min, max) or n (%)

Characteristics	No anemia (*n* = 868)	Anemia(*n* = 1100)	*P*-value
Gender			< 0.001
Male	448 (51.6%)	465 (42.3%)	
Female	420 (48.4%)	635 (57.7%)	
Age(years)	51 (18,83)	54 (18,94)	< 0.001
BMI (kg/m²)	24.1 (15.1,41)	22.6 (12.2,44.9)	< 0.001
ASA class			< 0.001
Ⅰ	79 (9.1%)	55(5%)	
Ⅱ	609 (70.2%)	636(57.9%)	
Ⅲ	178 (20.5%)	398(36.2%)	
Ⅳ	2 (0.2%)	11 (1%)	
Previous abdominal surgeries			< 0.001
1	491 (56.6%)	477 (43.4%)	
≥ 2	377 (43.4%)	623(56.6%)	
Operation time (min)	228 (29,726)	285 (43,930)	< 0.001
Anesthesia time (min)	304 (57,766)	364 (99,1000)	< 0.001

### Impact of anemia on prognosis

Significant differences were observed between the two groups in baseline characteristics including age, gender, BMI, and history of previous surgery (all *P* < 0.05). Compared with the non-anemia group, patients with anemia were more likely to be female (57.7% vs. 42.3%, *P* < 0.05), were older (median 54 vs. 51 years, *P* < 0.05), had lower BMI values (22.6 vs. 24.1, *P* < 0.05), had a higher proportion of ASA class III–IV (37.2% vs. 20.7%, *P* < 0.05), and had a greater rate of previous surgery (≥ 2 procedures: 56.6% vs. 43.4%, *P* < 0.05). Additionally, the anemia group experienced longer operation time (285 min vs. 228 min, *P* < 0.05) and longer anesthesia time (364 min vs. 304 min, *P* < 0.05) Table 1.

The anemia group also showed a higher incidence of severe complications (10.8% vs. 5.8%, *P* < 0.05), prolonged duration of mechanical ventilation (*P* < 0.05), longer ICU stay (*P* < 0.05), extended postoperative hospital stay (18 days vs. 15 days, *P* < 0.05), and longer total hospital stay (31 days vs. 26 days, *P* < 0.05). There were 4 deaths (0.2%) within 24 h and 20 deaths (1.0%) within 30 days after surgery Table 2.

**Table 2:** Outcomes of patients with and without preoperative anemia data are presented as median (min, max) or n (%)

Characteristics	No anemia (n = 868)	Anemia(n = 1100)	*P*-value
Severe postoperative			< 0.001
Mild	817 (94.2%)	980 (89.2%)	
Severe	50 (5.8%)	119 (10.8%)	
Total hospital stay (days)	26 (7,202)	31 (7,278)	< 0.001
Postoperative hospital stay (days)	15 (3,180)	18 (0,240)^a^	< 0.001
Mechanical ventilation time (hours)	0 (0,319)	0 (0,905)	< 0.001
ICU length of stay (days)	0 (0,51)	0 (0,134)	< 0.001
30-day mortality, n (%)^b^	4 (0.5%)	16 (1.5%)	0.029

* Postoperative stay recorded as 0 days for 4 patients who died within 24 h postoperatively

^a^Four people died within 24 h after the operation, and the postoperative hospital stay was recorded as 0

^b^Four cases died within 24 h after the operation were excluded

Multivariate analysis indicated that Preoperative anemia is associated with increased with increased risks for following outcomes: increased risk of severe complications (OR:1.9; 95%CI:1.3–2.7;*P* < 0.05), prolonged mechanical ventilation duration (β: 7.320; 95% CI: 3.0–11.6; *P* < 0.05), extended ICU stay (β: 0.744; 95%CI: 0.4–1.1; *P* < 0.05), prolonged postoperative hospitalization (β: 3.156; 95%CI: 1.3–5.0; *P* < 0.05), and total hospitalization duration (β: 5.041; 95%CI:3.1–7.0; *P* < 0.05).Thus, preoperative anemia was identified as an independent risk factor for these adverse outcomes. Preoperative anemia was not correlated with mortality at 24 h or 30 days after surgery Table 3.

**Table 3:** The association between preoperative anemia and clinical outcomes

Outcomes	Univariate analysis		Multiple-factor analysis	
	*P*-value	difference value[anemia, No Anemia]	*P*-value	OR/β[95%CI]
Complications (CD grade ≥ III), n (%)	< 0.001	5%[10.8%,5.8%]	< 0.001	OR1.9[1.3,2.7]
Total hospital stay (days)	< 0.001	5[31,26]	0.000	β5.041[3.1,7.0]
Postoperative hospital stay (days)	< 0.001	3[18,15]	0.001	β3.156[1.3, 5.0]
Mechanical ventilation time (hours)	< 0.001	0[0–905,0–319]	0.001	β7.320[3.0, 11.6]
ICU length of stay (days)	< 0.001	0[0–134,0–51]	0.000	β0.744[0.4, 1.1]
30-day mortality, n (%)	= 0.029	1%[1.5%,0.5%]	0.124	OR2.4[0.8,7.6]

## Discussion

In this retrospective study of 1968 patients with RPTs, the preoperative anemia rate was 55.9%. This incidence is consistent with that reported by Clarissa P et al. [14] in patients undergoing gastrointestinal cancer surgery (55.5%), but significantly higher than the rate reported by Molla et al. [9] for non-cardiac surgeries (43.7%), and slightly lower than that described by Joseph P et al. [15] in esophageal cancer patients (64%).

The relatively high prevalence of anemia in RPT patients may be attributed to several pathophysiological mechanisms unique to these tumors:

1. RPTs invasion of blood vessels and the gastrointestinal tract can lead to overt or chronic occult blood loss. Additionally, the tumor itself and associated chronic inflammation may release cytokines that suppress bone marrow hematopoiesis, disrupt iron metabolism and utilization, and reduce red blood cell survival [16];
2. RPTs hypermetabolism, coupled with loss of appetite and gastrointestinal involvement or dysfunction, may result in deficient intake or malabsorption of essential hematopoietic nutrients such as iron, folate, and vitamin B12;
3. The deep and anatomically complex location of RPTs often complicates surgical resection, and a history of multiple previous operations may further contribute to a higher incidence of anemia[5];
4. Prior treatments such as chemotherapy may also inhibit erythropoiesis [17].

Patients with preoperative anemia exhibited distinct characteristics, including a higher proportion of females, older age, lower body mass index (BMI), and a greater prevalence of ASA class III/IV. These findings indicate that anemic patients generally present with poorer overall health and diminished organ functional reserve. Moreover, the anemia group had a higher rate of reoperations, as well as significantly longer operative and anesthesia durations. These observations suggest that preoperative anemia serves as a significant biological marker of compromised systemic health and often coexists with—and may synergistically amplify—other risk factors such as advanced age, underlying malnutrition, and increased surgical complexity. Together, these elements contribute to an elevated perioperative risk profile. Identifying these high-risk subgroups is conducive to conducting targeted screening and early intervention in clinical practice.

Although female patients exhibit a greater susceptibility to anemia [18], the present study demonstrated a lower risk of severe complications among females compared to males (OR = 0.6, 95% CI: 0.4–0.8, *p* < 0.05). This protective effect may be attributed to several factors:

1. Males are more prone to developing certain types of RPTs—such as liposarcoma and leiomyosarcoma—that display more aggressive biological behavior [19]. These tumors are often large, highly vascularized, deeply situated, and frequently invade blood vessels and organs, thereby substantially increasing the risk of chronic blood loss and acute intraoperative hemorrhage. Consequently, both the tumor and its treatment are more likely to initiate or worsen anemia of chronic disease.
2. Accumulating evidence suggests that estradiol exerts antitumor effects across multiple cancer cell lines. For instance, it has been shown to inhibit proliferation in MCF7 breast cancer cells[20], reduce invasiveness in B16F10 melanoma cells (though without affecting migration)[21], and decrease the incidence of skin cancer[22].Prior treatments such as chemotherapy may also inhibit erythropoiesis [17].

Anemia is particularly prevalent among older adults, with one Brazilian study reporting an incidence as high as 68.2% in individuals over 65 years old [23]. Elderly patients often present with atypical symptoms of anemia, which may be misinterpreted as signs of aging or chronic illness, leading to delays in diagnosis and treatment [24].

Therefore, in female and elderly RPTs patients undergoing surgery, anemia should be recognized as a critical concern warranting heightened clinical vigilance and prioritized intervention.

This study demonstrated a significantly higher incidence of severe postoperative complications among retroperitoneal tumor patients with preoperative anemia compared to those without anemia (*P* < 0.001). These findings are consistent with a meta-analysis by Musallam et al. [25], which included over 200,000 non-cardiac surgery patients and confirmed that anemia increases the risk of postoperative complications—a conclusion further supported by Adrienne B et al. [26] in the context of abdominal cancer surgery. Boumahd et al. [27] identified preoperative anemia as an independent risk factor for postoperative morbidity, reporting a 90% increased risk of severe complications in anemic versus non-anemic patients (OR = 1.90, 95% CI: 1.30–2.70, *p* < 0.001).

As highlighted by Clarissa P et al. [14], preoperative anemia is associated with increased postoperative complications in gastrointestinal cancer surgery. This may be explained by its frequent correlation with underlying chronic conditions—such as malnutrition, chronic blood loss, malignancy, autoimmune disorders, chronic infections, or trauma—each of which may independently elevate surgical risk. Anemia should thus be regarded not merely as an abnormal laboratory value, but as a critical clinical indicator of impaired nutritional status and diminished physiological reserve prior to surgery.

The pathophysiological mechanisms through which anemia contributes to postoperative complications are multifactorial:

1. The tumor microenvironment persistently releases pro-inflammatory cytokines, activating the ubiquitin–proteasome system, which promotes muscle protein catabolism while suppressing anabolism[28, 29];
2. Anemia-induced tissue hypoxia impairs mitochondrial oxidative phosphorylation, thereby compromising ATP production, energy metabolism, and wound healing[28];
3. Reduced oxygen-carrying capacity and impaired tissue oxygenation—exacerbated by intraoperative hemodilution—lead to accumulated oxygen debt, mitochondrial dysfunction, and decreased ATP synthesis, ultimately affecting the proliferation, migration, and function of cells essential to wound repair[30].

Given that radical resection remains the primary treatment for RPTs, and considering that 51% of patients in this cohort required reoperation due to local recurrence, repeated surgeries contribute to abdominal adhesions, sustained inflammatory responses, and disordered iron metabolism [31]. These factors collectively establish a distinct pathophysiology termed “surgery-related anemia,” underscoring the importance and urgency of optimizing preoperative anemia management—particularly in patients with recurrent RPTs.

This study demonstrated that preoperative anemia is associated with increased utilization of multiple perioperative resources, as evidenced by prolonged durations of mechanical ventilation, ICU stay, postoperative hospitalization, and total length of hospital stay. These findings are consistent with those reported by Stefan et al. [32] in a study of 23,348 patients undergoing colon surgery.

Zheng et al. further observed that preoperative anemia in esophageal cancer patients was associated with extended hospital stays, attributing this primarily to a higher incidence of postoperative fatigue syndrome—manifested as delayed ambulation and slower recovery of gastrointestinal function—which serves as a key contributor to prolonged hospitalization [33].

Anemia impairs oxygen delivery to end organs and reduces ischemic tolerance of vital tissues during hemorrhagic episodes, thereby increasing the requirement for intraoperative red blood cell transfusion. Transfusion, in turn, elevates the risk of infections; each unit of transfused red blood cells is associated with an approximately 13% increase in the risk of hospital-acquired infection [34].

Moreover, prolonged mechanical ventilation exhibits a time-dependent correlation with the development of ventilator-associated pneumonia (VAP), further amplifying infection risk and initiating a vicious cycle that extends hospital stay.

Collectively, these findings underscore the significant negative impact of anemia on both medical resource efficiency and patient recovery. Even mild anemia can contribute to extended hospitalization [35, 36].

Given the high prevalence of anemia among cancer patients and those undergoing neoadjuvant chemotherapy—which may be further exacerbated by treatment—assessment and management of anemia should be a central element of preoperative optimization in this population. Substantial evidence indicates that anemia is not only an independent risk factor for increased postoperative morbidity and mortality [6, 10], but is also significantly associated with reduced local control rates, shorter disease-free survival (DFS) and overall survival (OS), and diminished quality of life [6].

However, multivariate logistic regression analysis in this study did not identify preoperative anemia as an independent risk factor for 30-day postoperative mortality (Tables 1, 2 and 3), a finding that contrasts with the results reported by Yixu et al. [6]. This discrepancy may be largely attributable to the frequent coexistence in anemic patients of other adverse prognostic factors—such as advanced age, high ASA classification, and surgical complexity—rather than to a direct independent effect of anemia per se. Moreover, 30-day mortality often represents an endpoint of severe complications rather than an isolated outcome. The limited number of mortality events in this cohort may also have reduced statistical power, underscoring the need for larger studies to clarify the net effect of anemia.

Although the prevalence of anemia varies across oncologic surgeries, its detrimental clinical impact is well-established. Acute intraoperative blood loss is often compounded by cancer-related anemia of chronic disease (ACD), malnutrition, and chemotherapy-induced bone marrow suppression. Previous studies have reported that anemia contributes to increased morbidity and mortality in surgical oncology patients [37]. Furthermore, perioperative blood transfusion may not fully mitigate this risk and has been associated with increased infection rates and potentially higher tumor recurrence [38].

Given the high incidence of anemia in patients with retroperitoneal tumors (RPTs), there is compelling evidence to support the integration of preoperative anemia screening and management as a fundamental component of the perioperative pathway for this population. Once anemia is identified, it should be addressed in accordance with Patient Blood Management (PBM) principles, which involve actively investigating underlying causes and implementing targeted interventions with the goal of optimizing hemoglobin levels before surgery. Once anemia is diagnosed, the cause should be identified as soon as possible and targeted treatment initiated: Iron homeostasis may play a primary role in organ failure concerning the immune system [39].For iron deficiency anemia, oral or intravenous iron supplementation can be given; For chronic anemia or chemotherapy-related anemia, the combination of erythropoietin (ESA) and iron supplements can be considered for treatment. For nutritional anemia, folic acid, vitamin B12 and other nutrients should be supplemented. Studies have shown that preoperative correction of anemia can effectively reduce the need for perioperative blood transfusion, lower the risk of infection, promote postoperative recovery, and may improve the long-term prognosis of cancer patients [40]. In the future, multi-center randomized controlled trials need to be conducted to further verify the impact of preoperative anemia optimization management on perioperative outcomes and long-term survival of RPT patients. Effective PBM not only strives to reduce the need for allogeneic blood transfusions and their associated risks but also aims to enhance tissue oxygen delivery and overall physiological reserve. Ultimately, these measures are intended to decrease the incidence of postoperative complications, shorten recovery time, reduce ICU admissions and duration of mechanical ventilation, and lower overall healthcare costs. Well-designed randomized controlled trials (RCTs) are urgently needed to further evaluate the precise value of perioperative anemia management in improving surgical safety and long-term survival outcomes.

## Study limitations and future directions

This single-center retrospective study did not perform stratified analysis based on pathological types of retroperitoneal tumors (RPT) or severity of anemia, which may introduce confounding and selection biases. Although demographic differences between the two patient groups were adjusted in multivariate analysis, residual confounding factors remain. Potential biases in baseline characteristic representativeness, standardized clinical procedures, and regional generalizability of conclusions could limit the study’s applicability to broader populations or other healthcare settings. While the World Health Organization (WHO) hemoglobin threshold was used for anemia definition for clinical convenience, it did not differentiate specific types (e.g., iron deficiency anemia, chronic disease anemia, renal anemia). Due to data integrity limitations in retrospective studies, we were unable to systematically collect iron metabolism indicators (e.g., serum iron, ferritin, transferrin saturation), vitamin B₁₂, folic acid, or erythropoietin levels, making stratification by anemia etiology unfeasible. Future prospective studies incorporating etiological classifications of anemia are essential to elucidate differential prognostic impacts across anemia types and provide evidence for personalized treatment strategies.

Retrosperitoneal tumors exhibit complex pathological diversity. This study’s failure to stratify by specific pathological subtypes may have obscured the mechanisms underlying anemia development and its impact on outcomes across different tumor types. Additionally, the absence of systematic evaluation of long-term patient outcomes (including survival rates and quality of life) hinders comprehensive assessment of preoperative anemia management’s practical value. The findings primarily highlight the correlation between anemia and perioperative outcomes in retroperitoneal tumor patients. To further validate these clinical implications, multicenter prospective studies or randomized controlled trials with systematic long-term follow-up analysis are required.

## Supplementary Information

Supplementary Material 1.

## Data Availability

The raw data supporting the conclusions of this article will be made available by the authors upon request and can only be shared anonymously. This restriction is due to hospital policies, which prohibit data sharing without clearly deﬁned purposes. Data transfer agreements must be in place before data can be.

## Abbreviations

95%CI: 95% Confidence Interval
WHO: World Health Organization
Hb: Hemoglobin
BMI: Body Mass Index
ASA: American society of anesthesiologists
EPO: Erythropoietin
LOS: length of stay
RPTs: retroperitoneal tumors
OS: overall survival
DFS: disease-free survival
RCT: Randomized Controlled Trial
