# Supplementary material for: Impact of preoperative anemia on postoperative rate of severe complications and short-term clinical outcomes after retroperitoneal tumor resection
Source: BMC Anesthesiol. 2025 Dec 27;26:91. doi: 10.1186/s12871-025-03573-2 (PMC12874748; doi:10.1186/s12871-025-03573-2)
Supplement: Supplementary file 1 — Supplementary Material 1. [file 12871_2025_3573_MOESM1_ESM.docx]

**Supplementary Table 1- Surgical Complications**

| Grade | Definition |
| --- | --- |
| Grade 1 | Any deviation from the normal postoperative course that does not require pharmacological treatment or surgical, endoscopic, or radiological interventions. Allowed therapeutic regimens include: antiemetics, antipyretics, analgesics, diuretics, electrolytes, and physiotherapy. This grade also includes wound infections opened at the bedside. |
| Grade 2 | Requires pharmacological treatment with drugs other than those allowed for grade 1 complications. Includes blood transfusions and total parenteral nutrition. |
| Grade 3 | Requires surgical, endoscopic, or radiological intervention |
| Grade 3a | Intervention not under general anesthesia |
| Grade 3b | Intervention under general anesthesia |
| Grade 4 | Life-threatening complication (including CNS complications)* requiring ICU management |
| Grade 4a | Single organ dysfunction (including dialysis) |
| Grade 4b | Multi-organ dysfunction |
| Grade 5 | Death of the patient |
| Suffix “d” | If a patient experiences a complication at the time of discharge, the suffix “d” (for“disability”) is added. This indicates that follow-up is required to fully evaluate the complication. |

*Intracranial hemorrhage, ischemic stroke, subarachnoid hemorrhage; excluding transient ischemic attacks.

**Supplementary Table 2-Clinical Examples of the Clavien-Dindo Classification System**

| Grade | Organ System | Definition |
| --- | --- | --- |
| 1 | Cardiac | Atrial fibrillation that resolved after correction of potassium imbalance |
|  |  | Respiratory |
|  |  | Neurological |
|  |  | Gastrointestinal |
|  |  | Renal |
|  |  | Other |
| 2 | Cardiac | Rapidly responsive arrhythmia requiring beta-blockers for heart rate control |
|  |  | Respiratory |
|  |  | Neurological |
|  |  | Gastrointestinal |
|  |  | Renal |
| Other | Wound infection treated bedside but requiring antibiotics due to cellulitis |  |
| 3a | Cardiac | Bradycardia requiring pacemaker implantation under local anesthesia |
|  | Neurological | Refer to Grade 4 |
|  | Gastrointestinal | Biloma after hepatectomy requiring percutaneous drainage |
|  | Renal | Ureteral stenosis after renal transplantation treated with stent placement |
| 3b | Other | Non-infected open wound sutured in the operating room under local anesthesia |
|  | Cardiac | Cardiac tamponade after windowing procedure, post-cardiac surgery |
|  | Neurological | Refer to Grade 4 |
|  | Gastrointestinal | Anastomotic leakage requiring relaparotomy and low rectalostomy |
|  | Renal | Ureteral stenosis after renal transplantation requiring surgical revision |
|  | Other | Wound dehiscence with evisceration |
| 4a | Cardiac | Heart failure resulting in low-output syndrome |
|  | Respiratory | Respiratory failure requiring intubation |
|  | Neurological | Ischemic stroke / Intracerebral hemorrhage |
|  | Gastrointestinal | Necrotizing pancreatitis |
|  | Renal | Renal insufficiency requiring dialysis |
| 4b | Cardiac | Same as 4a, but combined with renal failure |
|  | Respiratory | Same as 4a, but combined with renal failure |
|  | Neurological | Ischemic stroke / intracerebral hemorrhage accompanied by respiratory failure |
|  | Gastrointestinal | Same as 4a, but with hemodynamic instability |
|  | Renal | Same as 4a, but with hemodynamic instability |
|  | Cardiac | Cardiac insufficiency after myocardial infarction (4a·d) |
| d |  | Respiratory |
|  |  | Neurological |
|  |  | Gastrointestinal |
|  |  | Renal |
|  |  | Other |
| TIA, transient ischemic attack. | | |
